# The Effects of Intensive Rehabilitation Combined with Thiamine Treatment on Cognitive Recovery in a Case of Non-Alcoholic Wernicke–Korsakoff Syndrome

**DOI:** 10.3390/neurolint16010018

**Published:** 2024-02-14

**Authors:** Cinzia Palmirotta, Gilda Turi, Serena Tagliente, Michele Pansini, Stefania De Trane, Gianvito Lagravinese

**Affiliations:** 1 Istituti Clinici Scientifici Maugeri IRCCS, Laboratory of Neuropsychology, Bari Institute, 70124 Bari, Italy; cinzia.palmirotta@icsmaugeri.it (C.P.); gildaturi0@gmail.com (G.T.); serenatagliente.psi@gmail.com (S.T.); 2 Department of Radiology, Oxford University Hospitals NHS Foundation Trust, Oxford OX3 0AG, UK; michele.pansini@eoc.ch; 3 Clinica Di Radiologia EOC, Istituto Di Imaging Della Svizzera Italiana (IIMSI), 6900 Lugano, Switzerland; 4 Istituti Clinici Scientifici Maugeri IRCCS, Neurorehabilitation Unit of Bari Institute, 70124 Bari, Italy; stefania.detrane@icsmaugeri.it

**Keywords:** Wernicke–Korsakoff encephalopathy, rehabilitation, thiamine, cognitive deficit

## Abstract

Wernicke–Korsakoff Syndrome (WKS) is a severe neurological disorder resulting from thiamine deficiency, commonly associated with alcohol consumption but also stemming from dietary imbalances or other clinical conditions. Cognitive deficits, affecting memory and executive functions, pose a serious concern, with partial recovery often not complete. A 28-year-old woman underwent surgery for acute necrotizing hemorrhagic pancreatitis, leading to admission for post-acute intensive treatment due to prolonged bed rest syndrome. Clinical examinations revealed sensory–motor neuropathy, denervation in the active phase, mammillary body hyperintensity, and cognitive impairment. The patient exhibited poor orientation, lacked awareness of her clinical condition, and experienced impaired nonverbal memory, practical constructive issues, and planning difficulties—consistent with WKS. The patient received high-dose thiamine (300 mg TDS), coupled with daily physiokinesitherapy and occupational therapy. A final neuropsychological evaluation three months later showed substantial remission of executive and memory difficulties, improved spatial–temporal orientation, and enhanced awareness. The complex case required timely multidisciplinary intervention for accurate diagnosis and effective rehabilitation. The patient experienced rapid clinical improvement and cognitive recovery with high-dose thiamine and physiotherapy.

## 1. Introduction

Wernicke encephalopathy (WE) is a severe neurological disorder characterized by acute onset and results from prolonged thiamine (vitamin B1) deficiency due to insufficient intake or malabsorption. Thiamine is a vitamin involved in the metabolism of carbohydrates, fats, amino acids, glucose, and alcohol and plays a central role in ensuring the perfect functionality of the cells of the central and peripheral nervous system and of the myocardium [1].

Alongside symptoms such as drowsiness and dizziness, EW is characterized by a particular triad of neurological symptoms such as ataxia, nystagmus, and cognitive–behavioral impairments [2]. These include confusion, spatial disorientation, apathy, memory, and concentration difficulties [3].

The prevalence of EW is between 0.6 and 2%, and, as revealed by autopsy studies [2], this disease is often under-diagnosed, as the characteristic triad of symptoms appears in only 10% of patients, while in most cases, it presents clinically as a non-specific confusional state [4]. This makes the diagnosis complex and can lead to an aggravation of the clinical picture, to coma, and finally to death, the incidence of which varies from 10 to 20% [5].

If not treated promptly, EW can progress to Korsakoff Syndrome (KS), a form of anterograde and retrograde amnesia with confabulations. KS manifests itself mainly with an important impairment of declarative memory and in its components of episodic memory, relating to events accompanied by the time and place in which they occurred, and semantic memory, relating to concepts, symbols, and general knowledge about the world. In each of these subdomains, anterograde memory processes are typically more commonly impaired than retrograde memory processes [6]. Memories from the earliest years of life are usually better preserved than more recent ones.

Confabulations are a hallmark symptom of this disorder, as patients tend to create fuzzy, imaginary accounts of events they cannot recall or recall real memories that are out of context and out of order in a timeline. Impairment of key executive processes, including inhibition of response, updating of working memory, scheduling, and shifting, can also be observed. A lack of insight into the disease (anosognosia) is evident, as patients with KS do not seem to be aware of their own cognitive deficits. Together with the latter, KS is also characterized by apathy, emotional instability, and sudden personality changes [6].

KS does not necessarily occur as a consequence of WE, but both are often considered in the literature as a single disorder, termed Wernicke–Korsakoff Syndrome (WKS) [7]. Neuroimaging techniques generally show signal hyperintensity in the mammillary bodies, periaqueductal gray matter, thalamus, and colliculi, but lesions may also involve the pons, cerebellum, and hippocampus [8,9].

WKS is usually caused by excessive alcohol consumption; more rarely, it can instead be found following repeated episodes of vomiting accompanied by severe weight loss [10] and malabsorption syndrome, as in the case of acute pancreatitis. In the case of non-alcoholic WKS, the recovery of symptoms can be partial [11]. In particular, the remission of cognitive deficits is not complete, and often, these deficits tend to persist over time [11].

The case of a 28-year-old Italian woman with WKS secondary to acute necrotizing hemorrhagic pancreatitis of biliary origin, who underwent rapid clinical improvement and recovery of cognitive and behavioral functioning following pharmacological treatment with high doses of thiamin and physiotherapy treatment, is reported below.

## 2. Case Presentation

On the 18th of October 2021, a 28-year-old woman was hospitalized following an episode of acute abdominal pain and vomiting. Parents reported that the patient had no relevant past medical history, no smoking or drinking habits, and no nutritional problems. She was right-handed, had 13 years of schooling, was employed, married, and had a child. Upon her arrival at the Accident and Emergency Department, analgesic and antiemetic treatment were infused with no response to symptoms. Clinical and instrumental tests revealed acute pancreatitis, leading to an urgent median laparotomy surgery with necrosectomy. Persistent symptoms and hyperpyrexia prompted another surgery involving viscerolysis, mesocolon raffia, and cholecystectomy performed in November. Upon stabilization, the patient was discharged from the Surgery Unit for admission to the IRCCS Maugeri of Bari for post-acute intensive hospital treatment due to prolonged bed rest syndrome. Based on the following clinical and neuropsychological records, a diagnosis of Wernicke encephalopathy [12] due to thiamine deficiency was established (see Table 1).

### 2.1. Clinical Assessment

Upon arrival at our unit, blood tests were performed, as well as an ECG and chest X-ray. A urine sample was also collected for microbiology. Blood biochemistry analysis revealed minor changes for liver function tests (AST 41 U/L, ALT 47 U/L, GGT 62 U/L), acute phase reactants (CRP 6.5 mg/dL, ESR 90 mm/h), and low hematocrit (29.1%) levels. Serum ammonia level was 61 mcg/dL. Sodium and potassium levels were normal (Na: 140 meq/dL, K: 4.0 meq/dL).

During the hospitalization, an electromyographic examination detected sensory–motor neuropathy in the lower limbs, greater sensory impairment, and denervation in the active phase. Neurological examination revealed bilateral upward beating nystagmus, bilateral convergence deficit, diffuse muscular hypotonia, moderate–severe tetraparesis prevalent in the lower limbs, deep tendon reflexes that were absent ubiquitously, and severe deficit of deep sensitivity found in all four limbs.

In light of these findings, the patient was transferred to the Acute Neurology Department at “DiVenere Hospital” of Bari in order to be further investigated. A diagnostic lumbar puncture was performed and indicated albumin-cytological dissociation leading to the initiation of a cycle of intravenous immunoglobulins (see Table 2).

**Figure 1:**
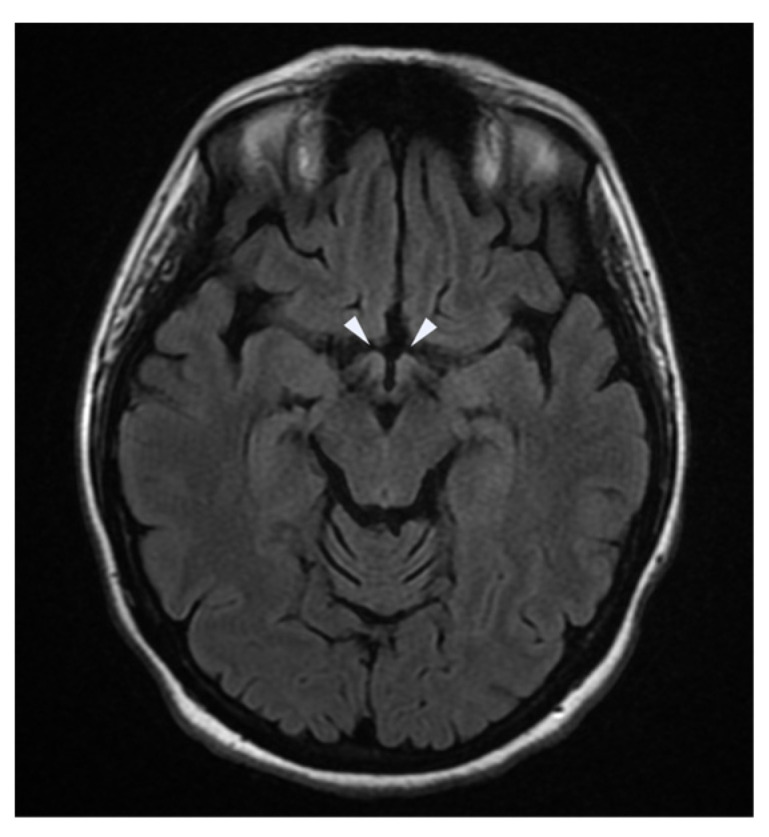
FLAIR axial view of the mammillary bodies.

**Figure 2:**
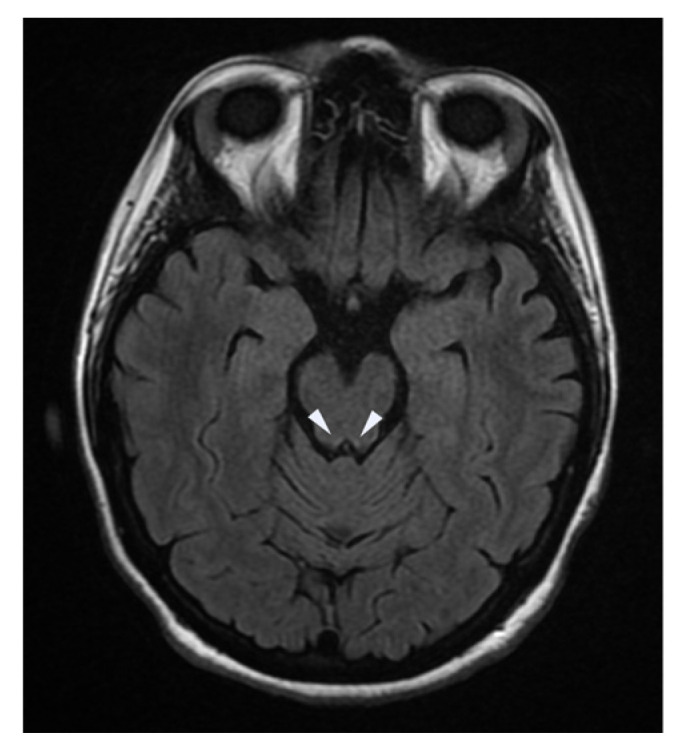
FLAIR axial view of the colliculi.

**Figure 3:**
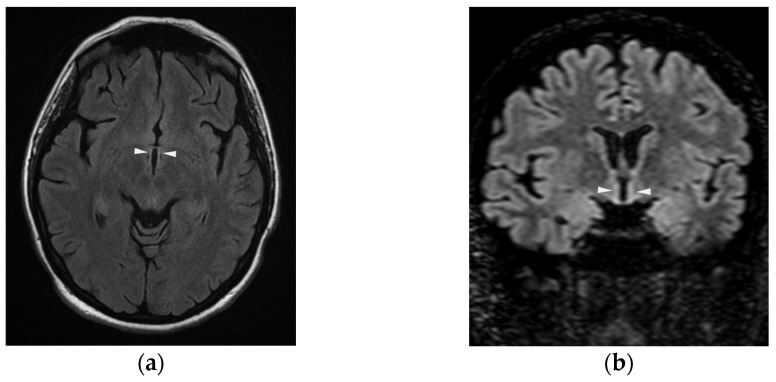
FLAIR views of periventricular region of the third ventricle in axial (**a**) and coronal (**b**) perspectives.

An MRI of the brain and spinal cord showed hyperintensity of the mammillary bodies (Figure 1), the region of the colliculi (Figure 2), and the periventricular region of the third ventricle (Figure 3). Blood tests showed severe hypovitaminosis (thiamine levels 3 μg/L). Following these assessments, the patient was admitted to the Neurorehabilitation Unit at the IRCCS Maugeri of Bari.

Figure 1 demonstrates a FLAIR axial MRI image with increased signal intensity in the mammillary bodies, reflecting the classic neuropathological changes of Wernicke encephalopathy, including neuronal loss, gliosis, and edema. The mammillary bodies are crucially implicated in the pathology of this disease, correlating with the clinical triad of confusion, ophthalmoplegia, and ataxia.

Figure 2 presents a FLAIR axial MRI image showing hyperintense signal alterations in the region of the colliculi, indicative of edema and potential gliosis commonly associated with Wernicke encephalopathy. These findings highlight the characteristic involvement of central midbrain structures in this condition.

Figure 3a showcases FLAIR MRI images in axial view. The arrows highlight symmetric hyperintense signals around the periventricular region of the third ventricle.

Figure 3b shows coronal view. The arrows spotlight symmetric hyperintense signals in the periventricular region of the third ventricle. These images underscore the characteristic involvement of thalamic and hypothalamic regions in Wernicke encephalopathy, reflecting the disease’s predilection for periaqueductal gray matter.

### 2.2. Neuropsychological Assessment

In order to investigate the cognitive–linguistic abilities, a neuropsychological assessment was conducted upon admission and upon discharge.

During the informal neuropsychological examination, the patient appeared alert, willing to talk, and cooperative but was easily fatigued and poorly oriented in time and space. The patient was not aware of her clinical picture. Difficulties of anterograde memory emerged while retrograde memory was preserved, except for events relating to the period of illness, surgical interventions, and previous hospitalization. During the interview, the patient’s mood appeared unstable and fluctuated from elated and cheerful to overwhelmed, sad, or frustrated. 

Spontaneous speech was characterized by fluent speech with slightly slowed prosody, consistent in form and content. The patient had a low voice tone, but their understanding of simple and complex sentences was good. The communicative modality was presented as very expressive and characterized by disinhibition, which can also be found at a behavioral level.

Established neuropsychological tests known for their reliability and widespread use were chosen to offer a comprehensive evaluation of neurocognitive function. Alternate forms of these tests were utilized during the post-training assessment to mitigate practice effects while preserving the essence of the measures employed. The evaluation encompassed various cognitive domains, including overall cognitive function, verbal and visual memory, attention, executive functions, visuo-constructive abilities, language, and functional assessment (see Table 3). Global cognitive functioning was assessed using the Mini-Mental State Examination [13]; short- and long-term memory were evaluated using the Rey 15-Word Test [14] and the Rey–Osterrieth Complex Figure Test [15], respectively; visuospatial short-term memory was assessed using the Corsi Block-Tapping Test [16]; verbal short-term memory was assessed using the Digit Span Test [17]; spatial learning was evaluated using the Corsi Supra Span Test [18]; sustained attention was measured using the Trail-Making Test Part A [19]; divided attention was assessed using the Trail-Making Test Part B [19]; working memory was evaluated using the Digit Span Backward Test [17] and the Corsi Span Backward Test [17]; interference sensitivity and inhibition of automatic responses were assessed using the Stroop Color and Word Test [20]; phonemic and semantic word retrieval were evaluated using the F-A-S Test and Semantic Fluency Test [21]; categorical thinking and executive functions were assessed using the Weigl Sorting Test [22]; and constructive praxis was evaluated using the Rey–Osterrieth Complex Figure Copy Test [15]. To detect the presence/absence of depression or anxiety symptoms, two self-report questionnaires were administered: the Beck Depression Inventory II and the Beck Anxiety Inventory [23]. The scores of assessment and re-test are displayed in Table 3.

### 2.3. Pharmacological Treatment and Rehabilitation Program

During hospitalization, the patient was administered thiamine (vitamin B1) in high doses (300 mg TDS). In addition, the patient was taking ursodeoxycholic acid, proton pump inhibitors, and neuropathic and neurotrophic pain medications daily. The rehabilitation treatment included physiokinesitherapy and occupational therapy and was divided into two daily sessions of 30 min each. The main objectives of this treatment were the improvement of the global muscle tone and tropism, the complete recovery of the articular range of the four limbs, and the gradual recovery of the proprioceptive sensitivity and balance; a 1:1 daily physiotherapy training was also performed in order to obtain the aided achievement of the sitting and standing posture.

## 3. Results

At the initial evaluation, the patient appeared anosognosic, confabulating, and with poor self-regulation skills. Cognitive functioning appeared to be characterized by mnemonic and executive deficits. Short-term, learning, and long-term difficulties (deferred recall) of verbal and spatial material were noted.

The patient was unable to recall a list of previously learned words after 15 min. In addition, the ability to recall non-verbal material was impaired. Even the performance in the copy test of this figure denoted considerable practical constructive planning and space organization difficulties. The tests revealed difficulties in tests of an executive nature, which require abstract reasoning skills, strategy, and cognitive flexibility. These data, together with the pathophysiological symptoms revealed by the neurological examination, showed a profile compatible with WKS. The final neuropsychological evaluation was carried out three months later. On informal examination, an improvement in spatio-temporal orientation and awareness of one’s clinical condition was visible. Mood was stable throughout the interviews and assessment. From the quantitative examination, a substantial remission of memory and executive difficulties was appreciated. Scores on tests assessing short-term, working, and long-term verbal and visuospatial memory skills were within normative ranges. In particular, an appreciable percentage improvement was noted in the performance of the spontaneous recall tests of word lists and complex figures, visuospatial learning, and figure copying. A notable increase in performance on attentional executive tests could also be noted (Table 3). As far as the motor aspects are concerned, the treatment resulted in a slight but gradual improvement in the strength of the upper limbs and trunk and the regression of the vertical nystagmus. However, at his resignation (10 March 2022), a marked paraparesis was more evident on the left, and tetrahypopallesthesia persisted.

## 4. Discussion

WKS is a serious disorder resulting from thiamine (vitamin B1) deficiency, primarily found in 90% of cases in individuals with alcohol dependence [24]. However, research indicates that this syndrome can also occur in other patient groups due to other causes, including bariatric surgery, cancer, hyperemesis gravidarum, anorexia nervosa, Crohn’s disease, schizophrenia, and depression, particularly in young and female patients [10].

In the presented case, following acute pancreatitis, the patient exhibited symptoms consistent with WKS, characterized by oculomotor and mental status alterations. Meeting DSM-5 criteria for WKS [12], the patient displayed severe anterograde and retrograde amnesia with confabulations, deficits in executive functions, poor initiative, various planning difficulties, organizational challenges, behavioral regulation issues, perseverations, rigidity, and anosognosia [25,26]. We can assume that fasting weight loss and the hypercatabolic state secondary to acute pancreatitis led to a critical malnutrition state with a severe thiamine deficiency.

From a neuromotor perspective, the patient demonstrated peripheral neuropathy, which is often associated with KS. These deficits are attributed to the loss of cholinergic neurons in the medial septum and malfunction of the Papez and frontocerebellar circuits, observable through MRI hyperintensity in the mammillary bodies [27]. Thiamine deficiency causes disruptions in acetylcholinergic synaptic transmission and reduces the metabolic activity of nerve cells, potentially leading to cell death [27].

Despite studies suggesting less prominent cognitive deficits in non-alcoholic WKS cases [28], the reported case highlighted severe mental and executive difficulties. Hospitalization and thiamine administration, either intravenous or oral, are essential for WKS treatment, preventing disorder progression and achieving neurological symptom remission within days. However, balance issues and confusion may persist for weeks or months, and memory loss may not fully resolve [5].

While some studies debate the correlation between thiamine doses and cognitive outcomes [24], the reported case indicated that high-dose thiamine, coupled with physiotherapy, led to nearly complete remission of neurological, mnemonic, and executive deficits, significantly enhancing cognitive functioning.

Through meticulous medical history collection, clinical evaluation, and consideration of nutritional status indicators, an accurate diagnosis was established, enabling the implementation of recognized therapeutic strategies.

The positive outcome achieved by the patient through physiotherapy underlines the crucial importance of timely intervention and comprehensive treatment, particularly emphasizing the significance of thiamine supplementation in cases of WKS. Given the complexity of WKS, a coordinated multidisciplinary intervention, comprising neurologists, physiatrists, neuropsychologists, and other specialists, was fundamental.

We suggest that physiotherapy complements drug therapy, facilitating functional and cognitive recovery.

This holistic approach ensures that all aspects of the patient’s condition are addressed, from medical management to nutritional support and rehabilitation. Encouraging and fostering the collaboration of different healthcare professionals within the hospital setting not only optimizes the quality of care delivered but also enhances patient safety and outcomes. Therefore, advocating for and prioritizing the implementation of multidisciplinary teams in healthcare settings is paramount to improving the overall quality and safety of patient care.

## 5. Conclusions

Wernicke–Korsakoff Syndrome is a clinical condition rarely associated with acute pancreatitis. It is manifested by ataxia, nystagmus, and cognitive impairment, particularly affecting memory and executive functions. The treatment of choice for this disorder is the administration of intravenous or oral thiamine. In the case presented, an improvement in cognitive functioning appears evident, assessed with a battery of neuropsychological tests, following therapy with vitamin B1 (300 mg three times a day) and physiotherapy treatment. It is essential for an ongoing collaboration among various healthcare professionals, including neurologists, medical physiatrists, psychologists, and rehabilitation specialists, to ensure a comprehensive and coordinated approach to Wernicke–Korsakoff patient care.

## Figures and Tables

**Table 1:** Diagnostic criteria of Wernicke–Korsakoff Syndrome.

**Diagnostic Criteria Wernicke’s Encephalopathy (DSM-5)** [12]
Presence of at least two of the following signs or symptoms: - Confusion or delirium. - Ophthalmoplegia (paralysis or weakness of eye muscles). - Ataxia (lack of muscle coordination). - Presence of dietary deficiencies, including thiamine (vitamin B1) or other nutritional deficiencies. - Additionally, there should be clinical evidence suggesting a causal relationship between the signs or symptoms and thiamine deficiency.
**Diagnostic Criteria Korsakoff’s Syndrome (DSM-5)**
Presence of all of the following: - Significant memory impairment that is not fully explained by other cognitive deficits. - Presence of confabulation (fabrication of stories) that is not due to delirium or another mental disorder. - Absence of clouding of consciousness or other cognitive deficits that would fully account for the clinical presentation. - Memory impairment should be confirmed by clinical evaluation and neuropsychological testing. - Evidence that the condition is not attributable to another medical condition, substance use, or medication side effects.

**Table 2:** Overview of the clinical and imaging features.

Clinical case Features Overview	
**Age**	28 years old.
**Gender**	Female.
**Medical History and Physical Examination**	No alcohol, smoking, or malnutrition history.
**Neurological Assessment**	- Bilateral upward beating nystagmus; - Bilateral convergence deficit; - Diffuse muscular hypotonia; - Moderate–severe tetraparesis prevalent in the lower limbs; - Deep tendon reflexes were absent ubiquitously; - Severe deficit of deep sensitivity in all four limbs.
**Laboratory Tests**	- RBC 25/μL; - WBC 1/μL; - Character: clear and colorless; - Glucose: 79 mg/dL; Protein: 65 mg/dL; - Blood tests showed severe B1 hypovitaminosis (thiamine 3 μg/L; range 32–95); - Blood liver function tests (AST 41 U/L, ALT 47 U/L, GGT 62 U/L); - Acute phase reactants (CRP 6.5 mg/dL, ESR 90 mm/h); - Low hematocrit (29.1%) levels; - Serum ammonia level: 61 mcg/dL; - Sodium and potassium levels were normal (Na: 140 meq/dL, K: 4.0 meq/dL).
**Cognitive Impairment and Behaviour**	- Drowsy, arousable, disoriented, anosognosic, confabulating, poor self-regulation skills; - Cognitive functioning appeared to be characterized by mnemonic and executive deficits. Short-term, learning, and long-term difficulties (deferred recall) with verbal and spatial memory.
**Brain Imaging**	MRI of the brain and spinal cord showed hyperintensity of: - Mammillary bodies; - Periventricular region of the third ventricle; - Midbrain tectum (superior and inferior colliculi).

**Table 3:** Neuropsychological assessment and final evaluation scores.

	Neuropsychological Assessment			Final Neuropsychological Evaluation			% Improvement
	P.G.	P.C.	P.E.	P.G.	P.C.	P.E.	
Global Cognitive Functioning							
Mini-Mental State Examination	27	25.59		29	27.59		7.41%
**Memory**							
Rey 15-Words—Instant Recall	**36**	**26.2**	**0**	47	37.2	3	30.56%
Rey 15-Words—Deferred Recall	**7**	**3.9**	**0**	13	9.9	4	85.71%
Figure of Rey–Osterrieth Deferred	**4**	**−1.25**	**0**	15	11	1	275%
Direct Span Courses	5	4.44	2	6	5.44	3	20%
Direct Digit Span	**4**	**3.44**	**0**	5	4.42	4	25%
Supra-Span Courses	**6.58**	**3.83**	**0**	16.68	8.93	1	153.50%
**Attention and Executive Functions**							
Trail-Making Test A	45	58	2	36	49	3	20%
Trail-Making Test B	153	198	1	72	117	3	52.94%
Trail-Making Test B-A	108	139	1	36	67	3	66.67%
Inverse Digit Span	**3**	**2.42**	**0**	5	4.42	4	66.67%
Inverse Span Courses	4	3.35	1	5	5.35	3	25%
Stroop Color and Word Test—Errors	1	2.75	2	0	0	4	/
Stroop Color and Word Test—Time	13.71	27.21	2	1.86	15.36	4	86.43%
Phonemic Fluency (F-A-S)	32	25.5	2	49	42.5	4	53.12%
Semantic Fluency	31	25	1	40	34	2	29.03%
WEIGL Test	**9**	**8**	**0**	13	12	3	44.44%
**Visuospatial skills**							
Figure of Rey–Osterrieth Copy	**13**	**10.25**	**0**	33	30.5	2	153.85%
**Mood**							
Beck Depression Inventory II	**12**		**Norm**	4		Norm	66.67%
Beck Anxiety Inventory	**6**		**Norm**	10		Norm	−66.67%

PG = raw score. PC = score corrected for age and level of education. PE = equivalent score: 0 = deficit (<5th percentile); 1 = borderline normal (5th–10th percentile); 2 = middle–lower (10th–25th percentile); 3 = medium–lower (25th–50th percentile); 4 = medium–upper (>50th percentile). N.E.: not executable. **Only a PE = 0 indicates a pathological performance**. Bold data highlight the pathological performances on cognitive tests.

## Data Availability

The patient’s personal data are protected according to the GDRP regulations of Italy and the EU.
